# Detection of HBV DNA in Cerumen and Sera of Hbsag Negative Patients with Chronic Hepatitis B Infection

**Published:** 2012-03-01

**Authors:** E Gholami Parizad, A Khosravi, E Gholami Parizad, K Sayehmiri, R Ranjbar

**Affiliations:** 1 Department of Microbiology, Ilam University of Medical Sciences, Ilam, Iran; 2 Department of Immunology, Ilam University of Medical Sciences, Ilam, Iran; 3 Department of Public Health, Ilam University of Medical Sciences, Ilam, Iran; 4 Department of Biostatistics, Ilam University of Medical Sciences, Ilam, Iran; 5 Molecular Biology Research Center, Baqhyatallah University of Medical Sciences, Tehran, Iran

**Keywords:** Hepatitis B, HBsAg, Serum, Carrier, Ear cerumen

Dear Editor,

Chronic carriers of hepatitis infection are living throughout the world mostly in the South East of Asia.[1][2][3][4][5] Recent studies have shown that some HBsAg negative individuals may develop some sort of chronic hepatitis B which is detectable employing some new laboratory diagnosis techniques such as molecular tests.[6][7] In human during decades, HBV had many mutations by which this virus has been able to escape from the immune system reactions so that HBV by far has changed its life style.[8][9] Considering such mutations into account, it seems necessary to employ some new diagnostic techniques particularly some standard laboratory tests to detect HBV in human fluids such as serum, cerumen, semen etc. ELISA is the routine standard test for diagnosis of HBV in medical laboratories using HBsAg but there is some limitation for this test[10][11] hence the quantitative PCR is recommended.[12] The current study was designed to evaluate the HBV either qualitatively or quantitatively in sera compared to ear cerumen as one non-invasive diagnosis method among both HBsAg negative and positive individuals in Ilam population using PCR, real time PCR and ELISA. PCR or real time PCR for HBV detection using cerumen was preferred as compared to serum as it is inexpensive, non-invasive, useful in epidemiological studies and more convenient to be performed in the clinic. Totally, 70 HBV patients whose infection was definitely detected as the control and 70 healthy individual without any history of HBV either clinically or documentary as the case group were randomly selected from those referring to the Blood Bank of Ilam, between January 2008 and January 2009. All sera were tested for HBsAg using ELISA while sera and cerumen samples were tested for the HBV DNA using PCR and real time PCR. ELISA was done according to the method described previously.[11] Mann-Whitney test was used to compare the median log HBV DNA in sera and cerumen. Association between the variables log HBV DNA in serum and cerumen was analysed using Spearman correlation. To indicate the importance of real time PCR compared to ELISA, Wilcoxon Signed Rank Test was used. Analysis was done using R software ver. 2.11.1. P value <0.05 was considered as significant throughout the study.

Results showed that only 2 individuals (2.89%) from the healthy people were HBsAg positive by ELISA, while 4.3% of participants in this group had HBV DNA by PCR and real time PCR indicating that 7% of individuals in case group were the chronic carriers of HBV (Table 1).

**Table 1:** Demographic and biochemical characteristics of all subjects among two groups.

**Variables**	**Groups**		***P *value**
	**Control (HBsAg+) (n=70)**	**Case (HBsAg-) (n=70)**	
Sex (male) (%)	38 (54.3)	41 (58.6)	0.60
Marital status (married) (%)	48 (82.9)	50 (71.4)	0.107
Age (Years) (Mean±SD)	32.4±6.5	29.2±5.9	0.003
HBsAg+	70 (100)	2 (2.9)	<0.001
Log Viral Load			
Serum HBV (Mean±SD)	12±4.8	0.75±2.9	<0.001^a^
Serum HBV (median and range)	10.9 (0-22.7)	0 (0-15.8)	<0.001^a^
Cerumen HBV (Mean±SD)	8.3±4.5	0.54±2.1	<0.001^a^
Cerumen (median and range)	8.6 (0-19.2)	0 (0-12.4)	<0.001^a^

^a^ P value was computed using Mann-Whitney test

PCR and real time PCR were found to be both superior to the ELISA particularly when cerumen and serum were both tested. The mean DNA copy/ml for cerumen and serum was 1.56×10(6) and 5.459×10(5) respectively. Kolciogu et al. who had studied 40 patients with chronic hepatitis B, reported that 12.5% of patients had HBV DNA in their creumen while 100% showed HBV DNA in their sera using real time PCR[13] which is not completely in agreement with our results in that the PCR and real time PCR results were similar. Goh, Eui-Kyung et al. had studied 30 patients who showed HBV DNA in their serum, and reported that 66.7% of those patients had HBV DNA in their cerumen as well.[12] The current study not only confirmed the ELISA results for all HBsAg positives; they all had HBV DNA in their sera and 87% of them showed HBV DNA in their cerumen, but also about 7 % of healthy individuals in case group had HBV DNA taking them into account as chronic carriers of HBV. There was a significant difference between the mean copy of log HBV DNA in serum and cerumen (p<0.001) favoring the higher mean copy/ml in cerumen than serum. The difference between PCR and real time PCR was not statistically significant but this difference was significant for ELISA and PCR (p<0.05) indicating the importance of real time PCR compared to ELISA.

Correlation coefficient between log HBV DNA in serum and cerumen of control group was 0.78 (p<0.001) while was 0.99 in case group confirming that cerumen can be used for both PCR and real time PCR procedures when detection of occult infection is the case even more effectively than serum. Another important finding is stressing on this point that HBsAg screening is not as reliable as molecular techniques in detecting HBV chronic carriers. On the other hand, it is merely important that exposure to the source of HBV infection is crucial in transmission of HBV, also not only with blood–derived products but also with cerumen and some body fluid like cerumen. As the this study could not cover the genetic variations of HBV DNA in cerumen and serum, further investigations are required to confirm the infectivity of human cerumen, as well as to determine the characteristics of HBV present in cerumen.
